# Practical Departments

**Published:** 1896-10-10

**Authors:** 


### PRACTICAL DEPARTMENTS.

### A NURSE'S INVENTIONS.

Nurse Ingram, of Barnstaple, has sent us particulars of two inventions of her own for sick-room use. One is an "extending foot-board," made of polished wood intended to be placed against the bottom of bed or couch to prevent slipping on the part of an invalid occupant. It consists of two flat pieces of wood, connected by a screw arrangement. One board rests against the bed, whilst the other can be extended at will to suit a tall or a short person. If the bed has but horizontal bars, detachable uprights are provided to keep the foot-board steady.

The second contrivance is for the benefit of bed-ridden and helpless patients or babies, to protect them from flies and gnats. It is a light, folding wooden frame, hooking at one end on to the top bar at the head of the bedstead, and resting by two jointed uprights on the bed at the other, taking in the upper half of the patient's body. Over this light frame a gossamer veil is thrown. Both appliances seem useful and practical, and we should recommend Nurse Ingram to communicate with the secretary of the Trained Nurses' Club, 12, Buckingham Street, Strand, with a view to their possible future exhibition among the nursing appliances on view there.

